# Exploring peer navigation and support in the quality of HIV care experiences of female sex workers in the Dominican Republic

**DOI:** 10.1186/s12913-021-07439-4

**Published:** 2022-01-11

**Authors:** Tahilin Sanchez Karver, Clare Barrington, Yeycy Donastorg, Martha Perez, Hoisex Gomez, Kathleen R. Page, David Celentano, Katherine Clegg Smith, Deanna Kerrigan

**Affiliations:** 1 Department of Prevention and Community Health, Milken Institute School of Public Health, George Washington University, Washington, DC USA; 2 Department of Health Behavior, Gillings Global School of Public Health, University of North Carolina, Chapel Hill, North Carolina USA; 3 HIV Vaccine Trials Research Unit, Instituto Dermatológico y Cirugía de la Piel, Santo Domingo, Dominican Republic; 4 Johns Hopkins University School of Medicine, Baltimore, MD USA; 5 Department of Epidemiology, Bloomberg School of Public Health, Johns Hopkins University, Baltimore, MD USA; 6 Department of Health, Behavior and Society, Bloomberg School of Public Health, Johns Hopkins University, Baltimore, MD USA

**Keywords:** HIV care continuum, Social support, Peer navigation, Quality of care

## Abstract

**Abstract:**

**Background:**

Despite evidence on peer navigation’s association with positive HIV outcomes, such as engagement in HIV care and antiretroviral therapy (ART) initiation, the mechanisms through which peer navigation may influence these outcomes have been less explored. The purpose of this study is to describe the role of peer navigation and support on enhancing the quality of HIV treatment and care services experienced by female sex workers (FSWs).

**Methods:**

Survey data was derived from a quantitative cohort (*n* = 211) of FSWs living with HIV in the Dominican Republic and complemented with data from two rounds of in-depth interviews (IDIs) from a qualitative subsample (*n* = 20 per round). Descriptive statistics and multivariable logistic regressions were used to explore the association between peer navigation and relational aspects of care and overall satisfaction of the quality of HIV treatment and care. Thematic analysis was employed to code and synthesize textual data from IDIs.

**Results:**

41.2% of the participants reported having had contact with a peer navigator in the last 6 months. Qualitative data revealed that peer navigation and support was instrumental in assisting FSWs linkage to HIV care after diagnosis, elevating FSWs’ ability to access more comprehensive clinical care facilities, and promoting agency by improving FSWs’ skills to more strategically and effectively engage with the clinic environment and health care providers. Peer navigation was positively associated with experiencing more respectful treatment by clinic staff (AOR: 6.65, 95% CI: 2.32–19.02), and greater satisfaction with overall HIV care services (AOR: 2.57, 95% CI: 1.77–3.74).

**Conclusion:**

Promoting the full integration of peer navigation into healthcare structures is a strategic approach to enhance the quality of HIV care experienced by FSWs and improve their HIV-related outcomes.

## Introduction

Peer navigation has proven to be an important strategy to improve HIV treatment and care outcomes, including within efforts to enhance engagement and retention in care, and support antiretroviral therapy (ART) initiation and adherence among people living with HIV (PLHIV) [1–6]. Through peer navigation, PLHIV are often better able to harness support to more effectively navigate through the HIV care continuum, often requiring a journey through disjointed healthcare structures [4].

Studies have highlighted the variations in the roles, responsibilities, and level of engagement of peer navigators within HIV treatment and care services [4, 6, 7]. Despite the variability in the implementation of peer navigation services, the effect of peer navigation has consistently been found to be instrumental in addressing the HIV epidemic and improving the quality of life for PLHIV across diverse settings and subgroups. For example, in South Africa, the use of peer navigators facilitated greater engagement in HIV care, ART adherence and enhanced HIV prevention practices by helping participants overcome self-stigmatizing views on their HIV status, which often prevented them from accessing care [1]. Another study conducted in the United States found significant associations between peer navigation and preventing declines in viral suppression [2]. Furthermore, in the Dominican Republic (DR), a study revealed the pivotal role of peer navigation through the implementation of a multi-level intervention [3, 8]. In this study, peer navigation was established to bring forth support and accompaniment to FSWs living with HIV, while also advocating for them [3, 8]. Results from this study found that peer navigation, along with individual counseling, sensitivity trainings for clinicians and a community mobilization strategy, improve ART adherence and consistent condom use among FSWs living with HIV [8]. Despite research surrounding the associations between peer navigation and HIV care outcomes, more evidence is needed to understand the mechanisms or pathways through which peer navigation influences HIV outcomes. Specifically, given findings on the critical role of clinic and provider dynamics on FSWs’ engagement in care, ART initiation and ART interruption [9–12], it is important to explore the influence that peer navigators may have in the quality of HIV care of FSWs as they progress through the HIV care continuum.

Given the recognition of peer navigation as a key strategic recommendation to meet UNAIDS 95–95-95 HIV epidemic goals by 2030 [7, 13], uncovering the pathways through which it influences the experiences of key populations, like FSWs, as they navigate through HIV care is essential as continued investments are being made to promote effective HIV prevention, treatment and care programs. Globally, FSWs are among the most vulnerable populations experiencing a disproportionate burden of HIV [3, 8, 14–21]. Furthermore, due to the intersection of identities and social conditions as it relates to their gender, occupation, and socioeconomic position, FSWs are susceptible to multiple forms of stigma and discrimination that only serve to heighten their susceptibility to HIV infection, and among those living with HIV, to poor treatment and care outcomes [22]. In the DR, FSWs continue to be a key target population in HIV prevention efforts as FSWs are nearly 5 times more likely to be living with HIV compared to other adults [8, 23]. Despite programmatic and intervention efforts, in the DR, the HIV epidemic goals continue to remain sub-optimal, with data suggesting that as of 2019 88% of PLHIV know their HIV status, 48% are receiving ART continuously, and 40% are virally suppressed [24]. Recommendations to address the HIV epidemic in the DR focus on targeting key populations, including FSWs, and integrating interventions and practices that foster their engagement and retention in care with the goal of reaching viral suppression, while also recognizing the importance of strengthening anti-discriminatory practices continuing to permeate healthcare structures [24].

The purpose of this study is to describe the role of peer navigation and support on enhancing the quality of HIV treatment and care services experienced by FSWs. We address this by (1) exploring the associations of peer navigation and overall satisfaction with HIV services and positive relational aspects of quality of HIV care, in particular improving patient-provider communication and respectful treatment by clinic staff; and (2) providing a more nuanced description of the level and type of engagement of FSWs with peer navigation and support as they engage with HIV treatment and care services by integrating qualitative and quantitative methodologies.

### Theoretical orientation

We conceptualized the role of peer navigation by drawing from Freire’s theory of critical consciousness [25, 26] to understand the effect that peer navigation may have on supporting the quality of HIV care received by FSWs. Freire’s theory on critical consciousness argues that people are often not aware of structural inequalities and as such cannot take the appropriate steps to combat oppressive systems that continue to perpetuate their inequality [25–27]. Freire maintained that it is important for people experiencing inequality due to oppressive norms and structures to “think critically about oppressive realities and challenge inequitable social conditions to reclaim their humanity” [27]. One method of achieving this is by generating a space for critical consciousness to grow through practices wherein people could become aware of their inequality through community empowerment strategies [28], such as through the use of peer navigation and support [29].

Among FSWs living with HIV, FSWs are exposed to a socio-cultural environment, which often limits their ability to navigate effectively through healthcare environments. FSWs often experience stigma and discrimination from the community and healthcare professionals [20, 24, 30], in addition, to experiencing their own self-stigmatizing views, which is often inundated with feelings of shame [20]. Among FSWs living with HIV, internalized stigma has been associated with missing ART treatment doses [31], while experiences of discrimination within healthcare structures, including harsh treatment, has been associated with healthcare avoidance [32]. Peer navigation and support could be a mechanism for combating these experiences and addressing the healthcare barriers that lead to suboptimal HIV outcomes among this population. Peer navigation provides an opportunity to focus on patients’ own lived realities outside of the healthcare environment, and thus, provides an opportunity to better assess the barriers that patients may be encountering as they navigate through healthcare structures and propose opportunities to meet those demands [29].

In our study, peer navigation and support are provided by current or former FSWs who have experience with HIV outreach, prevention, and support activities for FSWs living with HIV. Peer navigators are compensated with a monthly stipend for this role by the local research and intervention partner that collaborated in this study. The peer navigators are assigned a caseload of FSWs living with HIV and support their clients to access HIV and other services. We postulate that given peer navigators’ ability to provide culturally and linguistically appropriate assistance and support to FSWs living with HIV, while also challenging structural barriers within the healthcare system, peer navigation and support is a strategic approach to curtail the deficiencies within the healthcare system (e.g., poor treatment and communication by clinical care staff), and thus, better facilitate the pathway to viral suppression among this population.

## Methods

### Study design and parent study characteristics

This study was embedded within the NIMH-funded parent study “Stigma, cohesion and HIV outcomes among vulnerable women across epidemic settings” (R01MH110158) -a longitudinal observational cohort study being conducted in Iringa, Tanzania and Santo Domingo, DR- aiming to improve the health of FSWs and reduce the ongoing transmission of HIV by establishing the role of socio-structural and behavioral factors along the pathway to viral suppression. In each of these two settings, a cohort of approximately 200 FSWs living with HIV is being followed prospectively at 0, 12 and 24 months. In addition, as part of the parent study, a subsample of 40 women (*n* = 20 in Tanzania and n = 20 in the DR) from the cohort were invited to form part of a qualitative longitudinal cohort study aiming to explore the dynamic context of social cohesion and HIV and sex work related stigma among FSWs in relation to HIV services and outcomes. This qualitative sub-cohort is being followed prospectively and will complete a total of three rounds of annual in-depth interviews.

The current study drew from data from the second quantitative survey (at 12 months) in the DR from the parent study, while also utilizing the first two rounds of in-depth interviews from the qualitative subsample in the DR. Quantitative survey data was explored cross-sectionally. Meanwhile, we explored the in-depth interviews from a “repeated” [33] perspective to allow for a more in-depth exploration of peer navigation and support. As such, each interview presented an opportunity to enhance our understanding on the meaning and experiences conveyed by participants as it relates to their experiences with peer navigation and support.

### Study setting and participants

The DR cohort was based largely on an already established cohort of FSWs living with HIV in Santo Domingo through the *Abriendo Puertas* (Opening Doors) intervention [3, 8], with recruitment further enhanced through the assistance of peer navigators, key informants, and participants themselves. Eligibility criteria for participants included being at least 18 years of age, with a confirmed HIV-positive diagnosis, and having reported exchanging sex for money in the last month prior to enrollment. Women recruited for the study completed a socio-behavioral survey and blood was drawn for clinical laboratory tests at each study visit to assess HIV biological outcomes such as viral suppression.

### Data collection and measures

The current analysis uses the second round of data collection from the parent study (at 12 months post baseline) in the DR (*n* = 211), which took place between December 2018 and November 2019. Surveys were administered electronically by experienced interviewers utilizing a tablet in a private location. The survey examined a range of topics, including: demographic, occupational and HIV characteristics; patient-provider communication and dynamics; satisfaction with clinic and HIV services; and experience with peer navigation.

#### Relational aspects of quality of care

The survey was used to obtain two of the main outcomes of interest: patient-provider communication and respectful treatment by clinic staff in the HIV care environment. The patient-provider communication and trust was assessed through Galassi’s 15-item validated scale on Patient Reactions Assessment (PRA) [34]. PRA provides a measure for the perceived quality of the information given by the provider, affective behaviors of the provider and patient’s perceived ability to communicate with the provider [34]. This measure has been used in previous studies working with FSWs living with HIV in the DR [11, 35], and displayed a Cronbach’s alpha of 0.80. Respectful treatment by clinic staff was measured using a survey item assessing patient interaction with the clinic staff where they receive their HIV care. Specifically, this item measured feeling respected by clinic staff with answers on an ordinal scale ranging from 1 = always to 4 = rarely or never. This measure was dichotomized to indicate always being respected versus not always feeling respected by clinic staff.

#### Overall satisfaction with HIV care services

The last outcome of interest is overall satisfaction with HIV clinic services. Overall satisfaction with HIV care was assessed through a question inquiring participants’ rating of the services at the clinic where they receive their HIV care, with answers ranging from 1 = excellent to 5 = weak. This variable was dichotomized to indicate a rating of very good or excellent versus not very good or excellent. This measure on clinic satisfaction has been tested in this context (i.e., FSWs living with HIV in the DR) as part of the *Abriendo Puertas* program evaluation [35].

#### Peer navigation

The main independent variable of interest, peer navigation, was assessed utilizing 8 survey items, which were previously included as part of the *Abriendo Puertas* intervention evaluation in the DR [3, 8]. Participants were asked to report the level of engagement with peer navigators (including frequency of contact and type of engagement with peer navigators). Based on this information, we constructed a dichotomous measure to indicate whether participants have had any contact with a peer navigator in the past 6 months. Other variables related to peer navigation were used to provide a more nuanced description of the type and level of engagement participants reported having with a peer navigator.

### Statistical analysis

We first began exploring the data from the second round of the quantitative survey by conducting exploratory data analysis on outcomes of interest, relational factors of quality of care and satisfaction with HIV care, and peer navigation measures. Other socio-demographic, occupational, and HIV related variables were also explored as part of the analysis. Distributions of continuous variables were assessed noting particular parameters such as the mean, median, standard deviation, interquartile range (IQR) and range. Categorical variables were assessed through frequencies and proportions. Associations between the outcomes of interest, patient-provider communication, respectful treatment in HIV care environment and satisfaction with HIV services, and engagement with peer navigation were explored through Wilcoxon rank-sum and Chi-squares tests. Significant associations were then explored by utilizing a random-effects multivariable logistic regression. This type of regression was used to account for potential intra-cluster correlation among FSWs who access HIV care from the same facility. Within our sample (*n* = 211), there was a total of 11 different public and private clinics utilized by study participants. Results from this random-effects multivariable logistic regression are expressed in terms of adjusted odds ratio (AOR) and 95% confidence intervals (CI). All data management and analyses were conducted using the quantitative software Stata SE version 15.1 [36].

### Qualitative data collection, sample characteristics and analysis

The qualitative subsample (*n* = 20) was drawn from the main survey cohort from the DR utilizing a stratified purposeful sampling approach [37]. Using the existing data collected at baseline (at 0 months) participants were stratified into two groups: (1) those who were virally suppressed (less than 400 copies/mL) and (2) those who were not virally suppressed (400 or more copies/mL). The first round of qualitative in-depth interviews was completed between October and November 2018, while the second round was completed between December 2019 and February 2020. All in-depth interviews lasted approximately 60 min and were conducted in Spanish by two trained interviewers in a private location.

All interviews were transcribed and analyzed in Spanish, and select quotes were translated to English for the purpose of manuscript development. Data analysis focused on capturing the experiences of FSWs with peer navigation and support as they coursed through HIV treatment and care services. We began our analysis by reading each transcript thoroughly prior to coding to allow for a deeper immersion into the contextual realities of each participant. Next, interviews were analyzed using a thematic analysis approach [38], applying a combination of both inductive and deductive techniques. Freire’s theory on critical consciousness [25, 26] grounded the analytical process and provided a framework for what we expected to uncover in the analysis (for example, peer navigation/support and promoting agency). However, questions directly addressing the experiences with peer navigation and support were not included in the in-depth interview guides. As a result, many of the findings presented in this paper were based on emergent themes captured through the analytical exploration of the dynamics that contributed to the experience of quality of HIV care of FSWs. The findings presented were based on conversations wherein FSWs spontaneously spoke about their experience with peer navigation and support when addressing questions related to (1) first time linking to HIV care; (2) experiences with HIV treatment; (3) positive and negative experiences in the HIV clinic environment; and (4) recommendations for improving HIV care for FSWs. Throughout the coding and overall analytic processes, recurrent consultation with the research team allowed for a richer conceptualization and understanding of the findings surrounding peer navigation and support. ATLAS.ti version 8.4.4 [39] was used to managed the qualitative data.

### Ethical considerations

Approval for the current study was granted by the following Institutional Review Boards: Johns Hopkins University Bloomberg School of Public Health, the *Instituto Dermatológico Dominicano y Cirugía de Piel Dr. Huberto Bogaert Díaz* (IDCP) and the *Consejo Nacional de Bioética en Salud* (CONABIOS). All participants provided informed consent prior to participating in the study. Informed consent was reviewed with participants during each data collection activity. All data collected through quantitative and qualitative methods was de-identified to ensure anonymity of all participants.

## Results

### Sample characteristics

A total of 211 FSWs living with HIV participated in this study (Table 1). Participants had a median age of 41 years (IQR: 35–46), most had an educational attainment of primary education or less (61.1%), less than half reported currently having a partner (41.2%), and the vast majority were mothers (94.3%). The median number of years of sex work reported by participants was 19 (IQR: 12–26), and 34.6% of participants reported always or almost always engaging in substance use (alcohol or drugs) while meeting clients. 13.3% of participants reported experiencing any gender-based violence in the past 6 months. Regarding FSWs’ progress along the HIV care continuum, nearly all reported ever engaging with HIV related medical or clinical care (99.5%). Most women also reported currently being on an ART regimen (96.2%), with 79.3% among this sample also reporting being adherent to their ART regimen in the past 4 days. Overall, 76.2% of participants in the study were virally suppressed.

**Table 1:** Characteristics of quantitative sample of FSWs living with HIV (*n* = 211)

	Number	Percent
***Sociodemographic Characteristics***		
Age (median, IQR)	41	(35, 46)
Education		
Primary or less	129	61.1
Secondary or more	82	38.9
Relationship Status		
Without a partner	124	58.8
With a partner	87	41.2
Reported having children	199	94.3
***Occupational Characteristics and Risk Factors***		
Years in sex work^ (median, IQR)	19	(12, 26)
Experienced any GBV in the past 6 months	28	13.3
Substance use while working to meet clients		
Rarely or never	73	34.6
Sometimes	65	30.8
Always or almost always	73	34.6
***HIV care continuum***		
Received any HIV medical or clinical care	210	99.5
Currently on ART	203	96.2
ART Adherence in the past 4 days		
No	42	20.7
Yes	161	79.3
Viral suppression (< 400 copies/mL)		
Not suppressed	50	23.8
Virally suppressed (< 400 copies/mL)	160	76.2

^ *n* = 210

The sociodemographic characteristics of the qualitative subsample (*n* = 20) did not vary significantly from the quantitative cohort. However, FSWs’ progress along the HIV care continuum did vary from the quantitative cohort above. All FSWs in the qualitative subsample reported ever engaging with HIV related medical or clinical care, with 85.0% of FSWs reporting currently being on an ART regimen. Among those FSWs currently on ART, the vast majority reported being adherent to ART in the past 4 days (15/17; 88.2%).

### Engagement with peer navigation

Table 2 presents the results of FSWs’ engagement with peer navigation. 41.2% of FSWs reported coming in contact with a peer navigator about HIV related services and care in the past 6 months. Among participants who reported having any contact with a peer navigator, 68.3% of these had in-person contact with a peer navigator between 1 to 5 times in the past 6 months, whereas 31.7% of participants had in-person contact with a peer navigator 6 times or more. Similar distributions were found when asked about telephone contact with a peer navigator in the past 6 months.

**Table 2:** Engagement with peer navigation among FSWs living with HIV (n = 211)

	Number	Percent
Any contact with peer navigator about HIV related services and care in the past 6 months		
No	124	58.8
Yes	87	41.2
***Among participants in contact with a peer navigator:***		
In person contact with a peer navigator in the past 6 months*		
Between 1 and 5 times	56	68.3
6 times or more	26	31.7
Phone contact with a peer navigator in the past 6 months*		
Between 1 and 5 times	57	69.5
6 times or more	25	30.5
Has been reminded of clinic appointments by your peer navigator in the past 6 months*	54	65.9
Has been accompanied by your peer navigator to clinic appointments in the past 6 months	46	52.9
Type of accompaniment provided by peer navigator:		
HIV counseling and treatment	39	44.8
STI counseling	9	10.3
Has been accompanied by peer navigator to appointments not related to health in the past 6 months*	21	25.6

*n = 82

Most FSWs who had contact with a peer navigator were reminded of their HIV care appointments by their peer navigators (65.9%). Furthermore, over half of the women reporting having contact with a peer navigator also reported being accompanied by their peer navigators to a clinic appointment in the past 6 months (52.9%). For example, 44.8% of FSWs reported that their peer navigators accompanied them to an HIV counseling and treatment appointment, while 10.3% reported that their peer navigators accompanied them to a sexually transmitted infections (STI) counseling appointment. Some women (8.0%) also reported being accompanied by their peer navigators to other services or activities related to their HIV treatment and care including to collect medications or test results (data not shown in table). In addition, peer navigators also provided support outside of HIV care services. In fact, 13.8% of FSWs reported their peer navigators accompanied them to gynecological appointments to obtain a Pap smear test, while women also reported accompaniment by their peer navigators to violence counseling (3.45%), and family planning counseling (1.15%) (data not shown in table). Overall, over a quarter of FSWs engaging with peer navigators reported receiving accompaniment by their peer navigators to appointments not related to their health in the past six months (25.6%). These include to legal services, school appointments, talks or workshops for FSWs, and recreational activities.

### The role of peer navigation in linkage and retention in HIV care

Consistent with the quantitative results, less than half of the participants in the qualitative subsample spontaneously recalled experiences with peer navigation and support as they recounted their experiences with HIV treatment and care services. However, among the women who mentioned experiences with peer navigation and support, the pivotal role that peer navigation had in their experiences and the quality of their HIV care services was clear; specifically, the pivotal role that peer navigation and support had in ensuring that they were able to link to HIV care after confirmed HIV positive diagnosis. One participant recalled her experience linking to HIV care after finding out her diagnosis:

*“I found out [about my doctor] from [my peer navigator], [my peer navigator] gave me the talk… around the time I met [her], she lives with HIV, she tells me that she is going to take me to an [HIV] center to get me my medications, she went through everything with me, [my peer navigator], since they gave me my medications*.*”* [32 years old, 7 years living with HIV]

When describing her experience engaging with HIV care, another participant recalled how she is at her current HIV clinic because she was brought to the location by her peer navigator. This participant described *“just suffering”* for almost two years after her diagnosis, because she did not know how to get the HIV care that she needed. This participant reported finally engaging in HIV care through the help of a female friend who is also living with HIV and her peer navigator. She recalled *“I have a friend, but she doesn’t live there...That was the one who brought me here to the hospital, to the doctor, the one who brought me [to the doctor], that was [my peer navigator].”* The same participant explained how she has now become active in providing support, navigation and accompaniment to other women who are diagnosed with HIV just like the type of navigation and support that she received from her peer navigator despite knowing the risk of discrimination and stigma that she may face by members of her community. She explained:

*“That is why in [my community], they have said that I have HIV, because since I know that [in my community], one [person] has it, I go and look for [that person] and tell them 'I know where to take you, come let's go,' like the woman I took [to this clinic], I went and took [the person] to [my peer navigator], and [then] I took another woman to [my peer navigator] and I went with her to the doctor, so [the community] say 'that one has HIV', because when you have HIV, you go [to that clinic].”* [41 years old, 12 years living with HIV]

One participant also recalled her first engagement with HIV care services. She was able to access a HIV comprehensive care facility through her neighbor, who was also working as a peer counselor for FSWs and is a known activist among this community. When speaking about her experience, she recalled:

*“Because of her I am alive…When I got sick, then, she told me, ‘come here,’ I told her ‘come, I'm going to tell you [about my HIV diagnosis] that I know that you are going to help me,’…then she told me ‘don't despair, don’t worry I'm going to help you’ and she took me to the [the HIV clinic] with [my] doctor…[I trusted her] because I don’t know, she proved to be trustworthy, she proved to be very trustworthy.”* [49 years old, 7 years living with HIV]

### Promoting agency through peer navigation

Among participants who discussed experiences with peer navigation and support, there was a sense of agency or self-awareness of their own ability to make decisions about their HIV treatment and care. This level of self-awareness was aided by the information and support that peer navigators often provided to participants. One participant, who also serves as a peer navigator, promoted FSWs’ own level of awareness and control about their HIV treatment by discussing strategies for treatment adherence with them. She explained how she speaks to other FSWs living with HIV and shares her strategies for how they could also be adherent to their ART given their occupation. As she was explaining her strategies, she recalled letting other FSWs living with HIV know that *“you make a change, you are going to make a change, you sleep during the day”* and alerts them to reconsider when and how they take their medications given their own schedules. The same participant also described how she handles her own disagreements with clinic staff, utilizing her voice and activism to promote better quality of care services, while also encouraging other individuals living with HIV to better advocate for themselves. She recalled:

*“That day exploded, [participant recalling the day she initiated a protest because providers were not going to care for patients] they told me ‘shut up’ and [I said] ‘I'm not going to shut up.’[…]‘This mouth was put on me for three things…to eat, to talk and to suck’ […] Then the administration [from the clinic] said ‘what is happening here?’ [I said] we are protesting because here are all the patients from the morning and the ones from the afternoon just arrived and already the doctors…left and that is not possible.”* [53 years old, 38 years living with HIV]

Peer navigation and support was not limited to sharing information with participants about HIV treatment and care services, but also served as a linkage for women to connect to other groups that could provide additional support to FSWs, in particular. One participant recalled her sentiments related to knowing that she will need to be on an ART regimen for the rest of her life, and how through peer activities and support, introduced by her peer navigator, she surpassed any concerns that this may have imparted:

*“I was already prepared [to begin my ART medication], I no longer had any feelings [about having to take ART for the rest of my life], and to me that was normal, as I was already here [speaking of the Abriendo Puertas support group] and I had come here to listen to the talks every so often… I already knew about all of that [medication regimen and adherence].”* [41 years old, 12 years living with HIV]

In another example, one participant reflected on her own experience navigating the HIV care environment with the support of her peers, and it is that experience that has propelled her to desire a job wherein she serves to provide navigation and support to assist other women living with HIV. In the following, this participant explained her rationale:

*“[I would like to work and help more women who live with HIV] Yes, I would like to help people who don't have that…that support that I have had, that is, you always need that support, especially when you are recently diagnosed, but you always need that support, that is to always know that there is a person who thinks of you...that you have that [gives you a] helping hand, that even if you feel that you are falling there is a person who can lift you, even psychologically I would always like I mean, I want to,…, I want to help people who live with my condition and who may not have that person who can help them…there is a moment when you feel that [you are] alone, that you feel overwhelmed, that you feel sad, that you feel ... that you need at least one person to tell them about your problems…and even if you have a lot of family, even if you have many friends, it is not the same for you to sit with a person who is living the same [experience] as you or who has lived the same [experience] as you, who is going to understand the same, who is going to feel the same, who is going to know what you are saying.”* [34 years old, 14 years living with HIV]

### Promoting access to more comprehensive services

Through peer navigation and support, some participants were also able to better engage with HIV care services, such that women were able to have more information on which clinics or facilities to seek as they provide more holistic or comprehensive care. One participant recalled her experience trying to transfer to a clinic with help from her peer navigator:

*“[My peer navigator] wanted to take me to a [HIV care clinic] here in the capital…because in this [new clinic] they gave me my groceries, they do my analysis, I don't have to go out around the whole country, they do everything on one site.”* [32 years old, 7 years living with HIV]

Furthermore, when reflecting on her HIV care experience, another participant expressed that through her involvement in providing peer support as part of an organization for FSWs, she has been able to link women to more comprehensive care, which extends beyond HIV care. This participant expressed the following:

*“Look, for example here [at this site] they come and [they] do a study on you, if you come out with a disease, an infection, they also give you the medicine, which is a help because you do not have to buy it, it is a help. And there is much more. You can learn many things that can allow you to live by just coming here, the workshops they give here are things that allow you to survive economically because [you could learn] a craft…with that I can survive…”* [41 years old, 7 years living with HIV]

### The role of peer navigation in the HIV quality of care

Consistent with the qualitative findings, quantitative results further supported the contributing role that peer navigation has in the quality of HIV care. When exploring the associations between relational aspects of quality of care and overall satisfaction of HIV care by engagement with peer navigation (Table 3), we found two significant associations. First, most participants that reported engaging with peer navigation reported more respectful treatment by clinic staff. Specifically, 97.5% of FSWs who engaged with peer navigators reported always being treated with respect during care appointments, compared to 86.8% of FSWs who reported not engaging in peer navigation (*p*-value = 0.009). In addition, we found a significant association between satisfaction with HIV care services and peer navigation. Almost three-quarters of participants who had engagement with a peer navigator rated HIV care services as very good or excellent (72.8%) compared to just over half of participants who had no engagement with a peer navigator (52.1%; p-value = 0.003). Our results revealed no significant associations between peer navigation services and patient-provider communication.

**Table 3:** Relational aspects of quality of HIV care among FSWs living with HIV in the DR (n = 211)

	No Peer Navigation (***n*** = 121)		Peer Navigation (***n*** = 81)		***p***-value
	n	%	n	%	
The staff at the clinic where you get your HIV care always treats you with respect	105	86.8	79	97.5	0.009
Patient-Provider Communication (PRA Score) (median, IQR)	45	(43, 45)	45	(44, 45)	0.127
Overall, the services at the clinic where you get your HIV care are very good or excellent	63	52.1	59	72.8	0.003

We further explored the associations between engagement with peer navigation and always being treated with respect in the HIV clinic environment and the rating of HIV care services through random-effects multivariable logistic regressions. Our results indicate that after controlling for sociodemographic characteristics and years living with HIV, the relationship between the two outcomes of interest and peer navigation remained significant. As shown in Table 4, FSWs who reported engaging with a peer navigator have 6.65 times greater odds of also reporting always being treated with respect (95% CI: 2.32–19.02) in their HIV care environments compared to those with no engagement with peer navigation. In addition, compared to FSWs with no engagement with peer navigation, FSWs who reported engaging with a peer navigator had 2.57 times greater odds to rate the overall quality of HIV services received as very good or excellent (95% CI: 1.77–3.74).

**Table 4:** Associations between quality of HIV care measures and peer navigation (*n* = 201)

	Always being treated with respect at HIV care facility		Very good or excellent services at HIV care clinic	
	AOR	95% CI	AOR	95% CI
Age	1.04	0.96–1.13	1.02	0.98–1.06
Relationship: With a partner	0.93	0.56–1.55	1.68	0.86–3.31
Primary school or less	0.71	0.18–2.87	1.47	0.99–2.17
Years living with HIV	0.98	0.89–1.09	1.01	0.96–1.05
Any peer navigation	6.65**	2.32–19.02	2.57**	1.77–3.74

CI based on robust standard errors

** *p* < 0.01

The association between high levels of satisfaction with HIV care services and peer navigation was further confirmed through the qualitative findings. Specifically, when asked about recommendations that could improve the HIV care experience of FSWs, one recommendation was for clinics to establish navigation services for FSWs living with HIV. As explained by the following participant, navigation services could help women better understand and advocate for their own care:

*“The guidance, that there is always a person with you there, constantly, telling you and reading, inquiring because if you do not start inquiring and asking, [or have] the curiosity to ask, [then] you know that you will live all the time locked up and you will not learn anything.”* [37 years old, 12 years living with HIV]

## Discussion

The results of our study confirm the pivotal role that peer navigation and support have in improving the quality of HIV treatment and care services by addressing the structural and social barriers that hinder the experience of FSWs as they navigate through the HIV care environment.

Our study revealed the significant role of peer navigators in ensuring more respectful treatment by clinic staff, with FSWs who engage in peer navigation having greater odds of being treated with respect by clinic staff. This finding supports calls by UNAIDS to create more integrated services that dismantle discriminatory practices within the healthcare environments for PLHIV, including FSWs [24]. Having the support from peer navigators acts as a protective factor against experiencing poor treatment in healthcare facilities, which in part could be due to the accompaniment that peer navigators provide to their peers. This is important as disrespectful and abusive treatment by clinical care providers has been linked to HIV care appointments and disengagement in care [12]. Furthermore, our quantitative and qualitative findings support those by Alvis-Estrada and colleagues [7]: that peer navigators are providing support to FSWs in other areas outside of HIV care. For example, in our study, peer navigators provided accompaniment to family planning, violence counseling, and gynecological appointments, while they also linked FSWs to more comprehensive care with services that exceeded HIV care. This highlights the more comprehensive role that peer navigation play in the health and well-being of PLHIV.

As we stipulated using Freire’s theory of critical consciousness [25, 26], providing the space for culturally and linguistically appropriate exchanges allows individuals to address structural barriers that perpetuate inequality. Through peer navigation and support we argued that FSWs will be able to begin addressing the structural and social barriers that hinder their ability to receive quality HIV treatment and care. Our findings support this conceptualization of the role of peer navigation and support. In our study, having a positive HIV diagnosis was not sufficient to encourage FSWs to link to HIV care as confusion about how to course through healthcare structures, along with the deep-rooted fear and stigma that comes with a positive HIV diagnosis, serve to divert FSWs from assessing HIV treatment and care services. As a result, FSWs relied on the assistance of peer navigators to link to care and begin to understand the dynamics shaping the clinic environment. Furthermore, peer navigation and support provided a mechanism for women to establish their own control over their treatment and care, thus promoting greater agency among this often-stigmatized group. Peer navigation and support serve as an avenue for women to learn about best practices, where to find more comprehensive care services, and strategies for improving ART adherence and reaching viral suppression. Ultimately, FSWs were able to better navigate through the complex HIV care environments through established support generated by their peers. These findings corroborate the gains correlated with the utilization of peer navigation among PLHIV overall [1, 2, 6].

In addition to the UNAIDS call for the use of navigation services as a strategy for reaching the 95–95-95 HIV epidemic goals by 2030 [7, 13], FSWs themselves find the use of peer navigation as a promising strategy for improving their own experiences with HIV treatment and care services. Furthermore, data from this study also supports that FSWs who engage with peer navigators report better overall satisfaction with HIV care services. These findings are promising given that investments in peer navigation have been found to be cost-effective in addressing behavior among people with chronic illnesses [29], and given the support by FSWs themselves, this could be an approach that enhances the existing HIV care services to ensure the success of positive HIV outcomes among this population.

This study is not without limitations. Qualitatively exploring peer navigation and support was an emergent topic that stemmed from the exploration and analysis of the experiences of FSWs engaging in HIV care. As such, more in-depth exploration of the role of peer navigation and support and the healthcare environment’s views and perception about peer navigation was not considered in this study. However, we believe that its spontaneous emergence through the analytical process serves only to magnify the poignant role that peer navigation and support have in assisting FSWs to navigate through HIV care services and reach optimal HIV outcomes of ART adherence and viral suppression.

## Conclusion

Among this sample of FSWs living with HIV in the DR, peer navigation was found to be instrumental in addressing barriers to linkage and retention in care, promoting more comprehensive care, and enhancing more respectful HIV care environments. Integration of peer navigation into formal HIV care environments should be considered as an invaluable approach to improving the quality of HIV care services and attaining positive HIV outcomes among FSWs.

## Data Availability

The datasets used and/or analyzed during the current study are not available due to the sensitive nature of the information they contained, but are available from the corresponding author on reasonable request following appropriate ethical approvals.

## Abbreviations

AOR: Adjusted odds ratio
ART: Antiretroviral therapy
CI: Confidence Interval
CONABIOS: Consejo Nacional de Bioética en Salud
DR: Dominican Republic
FSWs: Female sex workers
HIV: Human immunodeficiency virus
IDCP: Instituto Dermatológico y Cirugía de Piel Dr. Huberto Bogaert Díaz
IDIs: In-depth interviews
IQR: Interquartile range
IRB: Institutional Review Board
MD: Maryland
NIMH: National Institute of Mental Health
PLHIV: People living with HIV
PRA: Patient Reactions Assessment
STI: Sexually transmitted infections
UNAIDS: Joint United Nations Programme on HIV and AIDS
USA: United States of America
